# Dental Jottings

**Published:** 1871-08

**Authors:** H. L. Sage


### DENTAL JOTTINGS.

BY H. L. SAGE.

### *Outward Inclination of a Lateral Incisor.*

Miss B., age 34; temperament sanguinous; teeth dense, yellowish-white, and rather long than short, called to consult in regard to the right superior lateral incisor, which from some cause not apparent, had within the previous year, changed position, having been forced outward, *i. e.* beyond the labial surfaces of the adjoining teeth, until its cutting edge had deviated one-quarter of an inch from a line with its neighbors. The movement was not in a vertical but in a horizontal direction. It had not at all elongated—was very firm, sound, natural in color, and well-formed. All of the other teeth were firm, regular and in good condition, in a well-developed arch, and the gums perfectly healthy. The first right superior molar was an exception—being quite loose and nearly ready to fall out, from the presence of tartar.

The gum in front of the displaced incisor, had not lost its attachment, and was also healthy.

On the cervico lingual portion of the tooth, was a slight deposit of tartar, and the connection with the gum was lost for about one-sixteenth of an inch, but the latter had not shortened or perceptibly receded. Over the end of the root, on the labial surface, some absorption of the alveolar process, had seemingly taken place, with a consequent falling in.

Was the absorption produced by the displacement of the tooth, or the displacement by the absorption?

Was the small deposit of tartar, and the slight loss of connection of tooth with gum, sufficient to cause the outward movement?

It was not forced outward by its antagonizing tooth, for the occlusion was such as to render such an event impossible. Neither does the slight loss of connection with the lingual portion of the gum, seem to be a sufficient cause.

Had the tooth been moved back to its original position, would it have become firm again at that age—34?—for though it was firm at the time, and probably had remained so during this *natural* process, a mechanical pressure would have loosened it.

Perhaps some may have fallen in with cases of a like nature, but I have seen nothing similar without a cause more apparent. What caused this outward movement (inclination) of the tooth, one-quarter of an inch, within so short a period, with no want of room, and all of the adjoining teeth and gums in a normal condition, let wiser ones determine. It must not be classed with cases so commonly met with, of teeth which become loose, elongate, and finally fall out.

### *Unequal Development of the two Halves of the Body.*

Having seen in the "*Cosmos*" for February 1871, among the selected articles, one entitled "Assymmetry of the two Halves of the Body," in which the writer speaks of the rarity of such cases, having found but one such as he describes recorded, (although in a foot note, allusion is made by Dr. Z., to the report of a case in the *American Journal of Medical Science*, for January 1871). I am inclined to contribute the following:

Miss —., age 19, has an irregular denture, the under teeth shutting outside of the upper ones, in front and on the left side. Left superior eye tooth projects anteriorly for want of room in the arch. The superior bicuspids and molars of the left side incline inward, and shut inside of the outer cusps of the inferior antagonizing teeth. These left upper side teeth all need forcing outward, and the jaw expanding, not only to make room for the left cuspid to fall back into place, but that the teeth of the same side may antagonize properly. She was advised accordingly. The teeth of the right side shut together perfectly, and the jaw is larger and more symmetrical than on the opposite side. The patient states that she is deformed—that the right side of her face is the fullest and largest—also that her right hand, as well as all the members of that side of the body, are larger than those of the other side. A casual observer would not notice it, however.

I should call it a more perfect development of the right side of the body than of the left, including the jaws, while a want of full development of the left side, extends also to those members, causing the teeth of that side to present for the lack of this development, a crowded and irregular condition, and an imperfect antagonism.

